# Environmental Factors Modulate Feeding Behavior of *Penaeus vannamei*: Insights from Passive Acoustic Monitoring

**DOI:** 10.3390/ani15142113

**Published:** 2025-07-17

**Authors:** Hanzun Zhang, Chao Yang, Yesen Li, Bin Ma, Boshan Zhu

**Affiliations:** 1 The Key Laboratory of Mariculture, Ministry of Education, Ocean University of China, 5 Yushan Road, Qingdao 266003, China; 2 Faculty of Information Science and Engineering, Ocean University of China, Qingdao 266100, China; 3 Beijing Aquatic Product Technology Promotion Department, Beijing 100176, China; 4 Behavioural Evolution Research Group, Max Planck Institute of Animal Behaviour, Konstanz 78467, Germany

**Keywords:** passive acoustic monitoring, aquaculture environmental factors, intelligent feeding system, feeding behavior, *Penaeus vannamei*

## Abstract

In recent years, passive acoustic monitoring (PAM) technology has significantly contributed to advancements in aquaculture techniques, system iterations, and increased production yields within intelligent feeding systems for *Penaeus vannamei*. However, current PAM-based intelligent feeding systems do not incorporate environmental factors into the decision process, limiting the improvement of monitoring accuracy in complex environments such as ponds. To establish a connection between environmental factors and the feeding acoustics of *P. vannamei*, this study utilized PAM technology combined with video analysis to investigate the effects of three key environmental factors—temperature, ammonia nitrogen, and nitrite nitrogen—on the feeding behavioral characteristics of shrimp, with a specific focus on acoustic signals “clicks”. The results demonstrated a significant correlation between the number of clicks and feed consumption in shrimp across different treatments, establishing this stable relationship as a reliable indicator for assessing shrimp feeding status. When water temperature increased from 20 °C to 32 °C, shrimp feed consumption showed an elevation from 0.46 g to 0.95 g per 30 min, with the average number of clicks increasing from 388 to 2947.58 and sound pressure levels rising accordingly. Conversely, ammonia nitrogen at 12 mg/L reduced feed consumption by 0.15 g and decreased click counts by 911.75 pulses compared to controls, while nitrite nitrogen at 40 mg/L similarly suppressed feed consumption by 0.15 g and the average number of clicks by 304.75. A rise in water temperature stimulated shrimp behaviors such as feeding, swimming, and foraging, while elevated concentrations of ammonia nitrogen and nitrite nitrogen significantly inhibited shrimp activity. Redundancy analysis revealed that temperature was the most prominent factor among the three environmental factors influencing shrimp feeding. This study is the first to quantify the specific effects of common environmental factors on the acoustic feeding signals and feeding behavior of *P. vannamei* using PAM technology. It confirms the feasibility of using PAM technology to assess shrimp feeding conditions under diverse environmental conditions and the necessity of integrating environmental monitoring modules into future feeding systems. This study provides behavioral evidence for the development of precise feeding technologies and the upgrade of intelligent feeding systems for *P. vannamei*.

## Simple Summary

In aquaculture, understanding how environmental factors influence shrimp feeding behavior is crucial for advancing intelligent feeding systems. This study utilized passive acoustic monitoring technology combined with video analysis to reveal that increasing water temperature significantly enhances feed consumption and feeding acoustic signals in *Penaeus vannamei*. Conversely, elevated concentrations of ammonia and nitrite nitrogen were found to suppress their feeding activity. Our analysis identified temperature as the prominent factor among the three environmental factors affecting shrimp feeding. This research quantifies the impact of common environmental factors on shrimp feeding acoustic signals for the first time, confirming that acoustic monitoring can effectively assess feeding status.

## 1. Introduction

In recent years, the growth rate of world aquaculture production has surpassed that of all other food production systems, emerging as a crucial pathway for ensuring future food security, driving poverty reduction, and achieving sustainable development [1]. Among these, the production growth of *Penaeus vannamei* has been particularly rapid due to a significant increase in the intensification of aquaculture [2], reaching 6.8 million tonnes in 2022 [1]. However, current feeding monitoring methods in shrimp pond aquaculture systems primarily rely on manual observation of feed trays. This subjective feeding strategy, characterized by delayed information and low accuracy, leads to severe feed waste, exacerbates water quality deterioration, and increases the risk of disease outbreaks, posing a bottleneck that limits the improvement of shrimp farming efficiency [3]. To further expand the scale of *P. vannamei* farming and enhance its intensification, there is an urgent need to develop high-precision, intelligent feeding systems with real-time decision-making capabilities to promote the transformation and upgrading of the shrimp farming industry [4].

Behavioral information provides real-time, efficient, and objective feedback on animal status, thereby enabling the development of precision intelligent feeding systems [5]. These behavior-based systems effectively contribute to improving aquaculture management and farming efficiency [6]. In the complex environment of pond aquaculture, passive acoustic monitoring (PAM) technology has demonstrated considerable potential in the intelligent feeding systems for shrimp pond farming due to its independence from visual limitations [7,8]. Previous studies have shown that the continuous “click” acoustic signals emitted by *P. vannamei* during feeding are closely related to feed consumption [9,10]. Quantifying and analyzing the characteristic parameters of these acoustic signals can assess shrimp feeding behavior and serve as pivotal data support for real-time monitoring of shrimp feeding status [11]. Currently, Australia’s AQ1 company has developed an intelligent feeding system for *P. vannamei* pond farming that utilizes PAM technology [12]. This system serves as the first successful application of PAM, providing a reference for precise feeding practices [13,14]. However, the practical implementation of PAM technology in aquaculture is still in its developmental phase and faces several challenges and limitations.

Fluctuations in environmental factors in pond aquaculture significantly affect the feeding behavior of shrimp [15]. For instance, ammonia nitrogen concentrations above 4 mg/L can lead to the enrichment of intestinal pathogens in shrimp, suppressing their appetite and reducing feeding behavior [16]. Exposure to a concentration of 20 mg/L nitrite nitrogen disrupts the intestinal mucosal structure of shrimp, further inhibiting their feeding [17,18]. Additionally, shrimp feed intake decreases with decreasing temperature within a specific range, and temperatures below 20 °C impair their immune defense [19,20]. Currently, automatic water quality monitoring systems can obtain real-time environmental parameters and have been preliminarily applied in pond aquaculture management [21]. However, most existing PAM-based intelligent feeding systems operate independently from water quality monitoring systems, resulting in a lack of information exchange between them. This design limitation, which excludes environmental factors, restricts the monitoring accuracy and feeding precision of feeding systems in the complex environment of pond aquaculture [22]. Therefore, there is an urgent need to establish a direct pathway from environmental data to behavioral and passive acoustic data, efficiently integrating multi-source information to enhance the response rate of feeding systems and improve the timeliness and scientificity of feeding decisions. To address this issue, this study selected temperature, ammonia nitrogen concentration, and nitrite nitrogen concentration as key environmental variables in aquaculture production. It analyzed the correlation between feed consumption and feeding acoustic characteristics and quantified the effects of environmental factors on the feeding behavior and acoustic characteristics of *P. vannamei*. Additionally, a comprehensive analysis of environmental factors, feed consumption, feeding behavior, and acoustic characteristics was conducted. The objective of this study is to provide behavioral insights that can inform the development of precision feeding technologies and the enhancement of intelligent feeding systems.

## 2. Materials and Methods

### 2.1. Animal Collection and Maintenance

The experiment was conducted from May to August 2022 at the Key Laboratory of Mariculture, Ministry of Education, Ocean University of China. *P. vannamei* used in the experiment were sourced from Yellow River Delta Marine Technology Co., Ltd., Dongying City, Shandong Province, China, with an average weight of 8 ± 0.32 g. Throughout the experiment, consistent natural seawater conditions were maintained, with a salinity of 30‰, a temperature of 26 ± 0.5 °C, an ammonia nitrogen concentration ≤ 0.02 mg/L, a nitrite nitrogen concentration ≤ 0.0013 mg/L, and a dissolved oxygen concentration of 5.5 ± 0.5 mg/L. The shrimp were acclimated for 7 days in glass aquariums (40.5 L, 45 cm × 30 cm × 30 cm) filled with natural seawater. The light–dark cycle was 12 h:12 h, with continuous aeration provided 24 h a day. Pelleted feed was provided ad libitum at 08:00 and 20:00 daily. One hour after each feeding, any remaining feed and excrement were promptly removed by siphon tubes, followed by replacing one-third of the water. The feed used in this experiment was 1.0 mm pellets (Haibo Feed Technology Co., Ltd., Yancheng, China) with a crude protein content of ≥42%.

### 2.2. Experimental Design

#### 2.2.1. Gradient Settings for Temperature, Ammonia Nitrogen, and Nitrite Nitrogen

Following acclimation, 660 individuals of *P. vannamei* in the intermolt period with intact appendages and normal activity were randomly selected and transferred to 11 experimental tanks (216 L, 80 cm × 60 cm × 45 cm), with 60 shrimp per tank. The experimental design comprised three independent sets conducted simultaneously, with the gradients and treatment settings for each set as follows: (1) Temperature gradient set: The shrimp were divided into three groups. The water temperature in two of the groups was adjusted at a rate of 2 °C per day to reach 20 °C and 32 °C, respectively, while the temperature in the third group remained at 26 °C. The ammonia nitrogen and nitrite nitrogen concentrations in all groups were consistent with those in natural seawater. (2) Ammonia nitrogen gradient set: The shrimp were divided into four groups. The control group used natural seawater with the original ammonia nitrogen concentration, while the other three groups had their ammonia nitrogen concentrations adjusted to 4 mg/L, 8 mg/L, and 12 mg/L using a 10 g/L ammonium chloride solution, with an increase of 2 mg/L every half-day. The temperature and nitrite nitrogen concentrations in all groups were consistent with those in natural seawater. (3) Nitrite nitrogen gradient set: The shrimp were divided into four groups. The control group used natural seawater with the original nitrite nitrogen concentration, while the other three groups had their nitrite nitrogen concentrations adjusted to 10 mg/L, 20 mg/L, and 40 mg/L using a 20 g/L sodium nitrite solution, with a gradual increase of 5 mg/L every half-day. The temperature and ammonia nitrogen concentrations in all groups were consistent with those in natural seawater. Once a group reached the target temperature, ammonia nitrogen concentration, and nitrite nitrogen concentration, the shrimp in this group were acclimated in the corresponding environment for 7 days with continuous aeration, and feeding conditions were maintained as described in Section 2.1. During the acclimation period, the levels of target environment factors in each experiment were detected and calibrated after daily water changes.

#### 2.2.2. Acquisition of Acoustic Feeding Signals and Calculation of Feed Consumption

**Figure 1:**
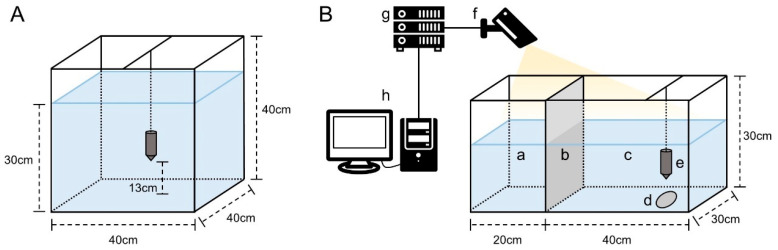
Audio acquisition system (**A**) and feeding behavior observation system (**B**). Note: isolation zone (a), partition (b), observation zone (c), feeding tray (d), hydrophone (e), infrared camera (f), switch (g), monitor and storage device (h).

Acoustic feeding signals from shrimp under different environmental conditions were collected using an audio acquisition system equipped with a hydrophone (Soundtrap 300 STD, Ocean Instruments, New Zealand) (Figure 1A). Following 24 h of starvation in the environmental treatment tanks, one shrimp was randomly selected from each tank and transferred to a transparent glass aquarium (40 × 40 × 40 cm) containing 48 L of seawater, with water quality conditions matching the corresponding experimental conditions. The hydrophone was positioned at the center of the aquarium. Aeration was discontinued after a 30 min acclimation. Subsequently, pellets equivalent to 5% of the individual body weight were introduced [10], and audio recording was started. The acoustic signals were continuously recorded at 48 kHz (16 Bits) with a hydrophone gain of 26 dB and sensitivity of −176.3 dB re 1 V/μPa. The recording was stopped after 30 min, and uneaten pellets were collected for feed consumption calculation. The collected residual feed was dried in an oven at 60 °C for 48 h to determine the dry weight. To ascertain the solubility loss rate, five groups of pellets (1.0 g each) were placed in experimental tanks without shrimp, and the remaining pellets were collected after 30 min, following the same processing procedure as described above. Feed consumption (FC) was calculated using the following formula:

(1) $$FC=\left(F_{0}-F_{r}\right)\times F_{1}$$

where $F_{0}$ is the amount of pellets provided (g), $F_{r}$ is the amount of pellets collected (g), and $F_{1}$ is the insolubility rate, calculated as the ratio of collected pellets to provided pellets in tanks without shrimp (i.e., $F_{1}=\frac{F_{r}}{F_{0}}$). Results were based on dry matter. Each environmental treatment was replicated with 12 shrimp for measurement.

The relationship between temperature and feeding is characterized by the Q10 coefficient, which represents the change in feeding acoustic signals for every 10 °C increase in temperature [23]. For the temperature gradient treatment groups, the number of clicks was used as a proxy to calculate Q10 values for the two temperature intervals of 20–26 °C and 26–32 °C. Q10 was calculated using the following formula:

(2) $$Q10={\left(\frac{R_{2}}{R_{1}}\right)}^{\frac{10}{T_{2}-T_{1}}}$$

where *R*_1_ and *R*_2_ denote the ratios of the number of clicks to the recording duration at temperatures *T*_1_ and *T*_2_.

#### 2.2.3. Video Recording of Feeding Behavior

The feeding behavior of shrimp under various environmental conditions was recorded using a feeding behavior observation system (Figure 1B). This system comprised a transparent glass aquarium (60 × 30 × 30 cm), a hydrophone, an infrared camera (DS-2CD864, Hikvision, China), and a monitor (233i, Philips, The Netherlands). The infrared camera recorded at a frame rate of 30 fps with a resolution of 1920 × 1080 pixels. The aquarium was divided into an isolation zone and an observation zone by a partition. The water quality conditions in the aquarium were consistent with the corresponding experimental conditions. After 24 h of starvation in the environmental treatment tanks, one shrimp was transferred to the isolation zone for a 30 min acclimation. Subsequently, pellets equivalent to 5% of the individual body weight were introduced on the feeding tray, and the partition was removed, with the position of shrimp at this moment defined as the initial location. Video recording started immediately and lasted for 30 min. Each environmental treatment was replicated with 12 shrimp for measurement.

### 2.3. Data Processing

#### 2.3.1. Audio Processing

Audio recordings from each treatment were transferred from the hydrophone to a computer using SoundTrap Host software (version 2.0.10, Ocean Instruments, New Zealand). Synchronization of the audio and video tracks was achieved by calibrating both systems to the same real-time clock prior to recording, followed by multi-track alignment in Adobe Premiere Pro (version 2022, Adobe, USA) using timestamp matching. Audio analysis was conducted using Raven Pro (version 1.6.5, Cornell Laboratory of Ornithology, USA) to generate waveform and spectrogram images (type: HANN; overlap: 50%; window size: 512). Extra noise, such as the sound of pellets hitting the water or shrimp accidentally bumping into the hydrophone, was manually removed from the recordings. According to the test results of environmental noise before the formal experiment, a filter was applied to eliminate low-frequency background noise below 1 kHz. During feeding, shrimp emitted “click” acoustic signals that appeared as distinct pulse waveforms in the audio recordings. An automatic pulse signal detection was performed using an energy detector with parameters set to a minimum occupancy of 90%, a signal-to-noise ratio (SNR) threshold of 10 dB, and a bandwidth frequency range of 3 to 48 kHz. Thirty click acoustic signals were randomly selected for further analysis of acoustic characteristics related to feeding activities. The analyzed parameters included the duration (ms), minimum and maximum frequencies (kHz), and frequency peak (kHz) of each pulse. Sound pressure level (SPL, in dB re 1 μPa) was used to quantify the intensity of the acoustic signals produced during feeding, representing the magnitude of sound energy. SPL was calculated as root mean square (RMS) derived from the power spectral density (PSD) using Matlab (version R2023a, MathWorks, USA) with the PAMGuide toolbox, employing an end-to-end calibration mode with a sensitivity setting of −176.3 dB. The calculation formula for SPL is as follows:

(3) $$SPL=10{log}_{10}\left(\frac{SPD\cdot ∆f}{p_{0}^{2}}\right)dB$$

where Δ*f* is the bandwidth (3–48 kHz) and *p*_0_ is the reference sound pressure (20 × 10^−6^ Pa).

#### 2.3.2. Video Processing

Video data were imported from the video recorder to a laptop computer (XiaoXinAir-14IIL 2020, Intel Core i5-1035G1, Lenovo, Hong Kong, China) via a solid-state drive (PS6, Lenovo, China). The swimming trajectory of shrimp was analyzed using EthoVision XT (version 14, Noldus Information Technology, The Netherlands). Initially, sampling points were manually marked on the shrimp. Detection parameters were set at a sampling rate of 8 frames per second, utilizing dynamic silhouette tracking as the detection method. Lost frame correction and smooth trajectory settings were enabled to enhance data accuracy. Trajectory smoothing was configured to average each sampling point with the preceding and following 10 samples. If the movement distance between consecutive sampling points was less than 3 cm, the latter point was retained at the position of the former. Conversely, if the maximum movement distance exceeded 20 cm within a sampling interval, the sampling point was designated as missing. Calibration of the scale was performed using a ruler, and the analysis and observation zones were manually delineated.

Based on the classification and definitions of feeding behavior in *P. vannamei* provided by Bardera et al. [24], the feeding process was categorized into feeding time, swimming time, and quiescent time. Each behavioral recording had a total duration of 30 min. Additionally, the time taken for the shrimp to first enter the feeding tray, defined as foraging time, was recorded as a separate parameter. Specific definitions for behavioral component classification are outlined in Table 1. Quantitative analysis of the recorded behavior videos was conducted using EthoVision XT 14, and statistical analysis was performed to calculate relevant feeding behavior parameters.

### 2.4. Data Analysis

One-way ANOVA was conducted to analyze the effects of different environmental factors on the feed consumption, number of clicks, SPL, and behavioral components of the shrimp. Multiple comparisons among groups were performed using Tukey’s test. Prior to ANOVA, the Shapiro–Wilk test was used to assess the normality of the data, and Levene’s test was used to check for homogeneity of variances. When necessary, data were transformed using the arcsine of the square root or logarithmic transformation. Spearman’s correlation analysis was used to examine the relationship between feed consumption and both number of clicks and SPL. The significance of linear regression coefficients was compared using *t*-tests, followed by multiple linear regression analysis to model the correlation between feed consumption and these acoustic variables. ANOVA, Tukey’s test, Levene’s test, and Spearman’s correlation analysis were performed using SPSS (version 27.0, IBM Corporation, USA). To compare the impacts of different environmental factors, redundancy analysis (RDA) was conducted. Temperature, ammonia nitrogen, and nitrite nitrogen were treated as explanatory variables, while the acoustic characteristics of feeding sounds and the duration of feeding behavior (in seconds) were considered as biological response variables. Detrended correspondence analysis (DCA) was conducted on the biological response variables to select the appropriate ordination model based on the length of the gradient of the ordination axis. The results showed that the longest gradient of DCA (1.15) was less than 2, indicating that RDA was suitable for analyzing [25]. Prior to RDA analysis, the biological response variable data were transformed using log (x + 1) to improve the comparability of variables with different units. All data in this study are presented as mean ± SD, and a significance level of *p* < 0.05 was used for all statistical tests. RDA analysis was conducted using Canoco software (version 5.0, Biometris, The Netherlands).

## 3. Results

### 3.1. Effects of Environmental Factors on Feed Consumption and Acoustic Feeding Signals of P. vannamei

**Figure 2:**
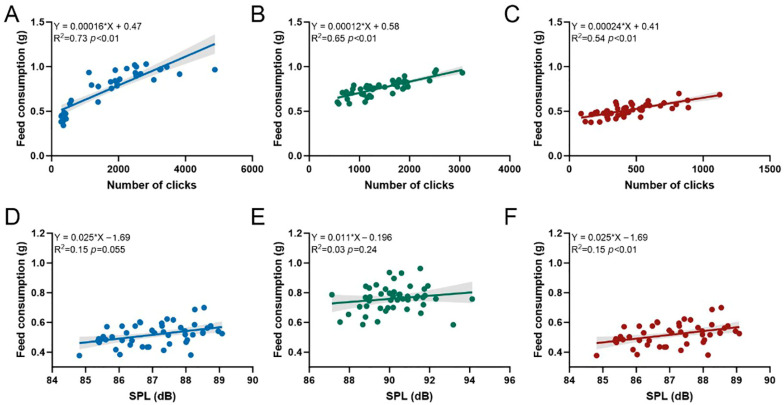
Relationships between feed consumption and both the number of clicks (**A**–**C**) and sound pressure level (SPL, in dB) (**D**–**F**) within 30 min after pellet feeding, under different temperatures, ammonia nitrogen concentrations, and nitrite nitrogen concentrations.

Under varying conditions of temperature (Figure 2A), ammonia nitrogen concentration (Figure 2B), and nitrite nitrogen concentration (Figure 2C), a significant linear relationship was observed between the feed consumption and both the number of clicks and SPL of *P. vannamei* during feeding (Table S1). The regression equations for each condition are as follows: for temperature, Y=0.00016×X+0.47 (R^2^ = 0.73, *p* < 0.01); for ammonia nitrogen, Y=0.00012×X+0.58 (R^2^ = 0.65, *p* < 0.01); and for nitrite nitrogen, Y=0.00024×X+0.41 (R^2^ = 0.54, *p* < 0.01). The linear relationship between the feed consumption and SPL was only significant under varying nitrite nitrogen concentrations (Figure 2E), with the regression equation Y=0.025×X−1.69 (R^2^ = 0.15, *p* < 0.01). No significant correlation was found between feed consumption and SPL under different temperature and ammonia nitrogen conditions (Table S1).

**Figure 3:**
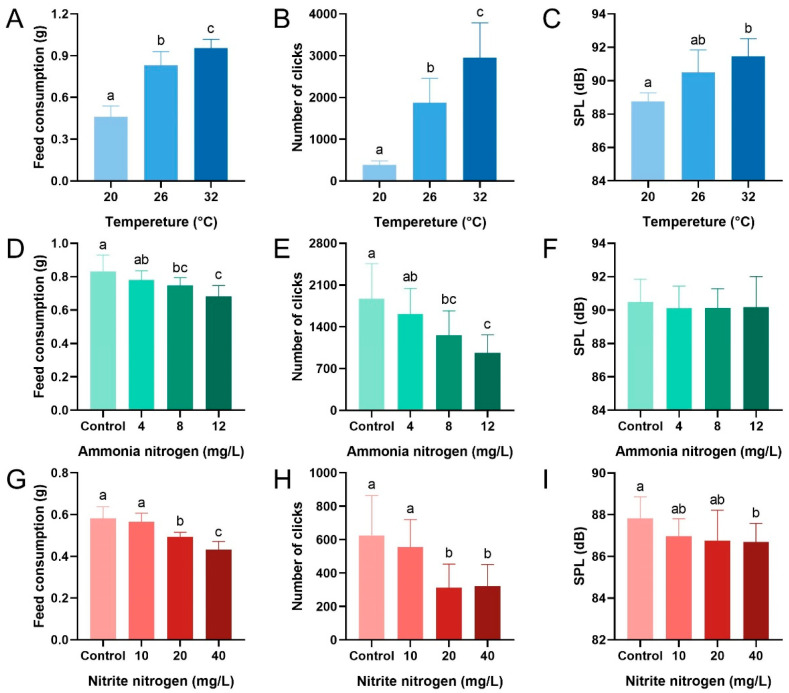
Differences in feed consumption, number of clicks, and sound pressure level (SPL, in dB) of *P. vannamei* within 30 min after pellet feeding, under varying temperatures (**A**–**C**), ammonia nitrogen concentrations (**D**–**F**), and nitrite nitrogen concentrations (**G**–**I**). Different lowercase letters indicate significant differences between treatment groups (*p* < 0.05).

Temperature significantly increased the feed consumption, the number of clicks, and SPL during feeding in *P. vannamei* (feed consumption: F_2,33_ = 119.693, *p* < 0.001; the number of clicks: F_2,33_ = 56.549, *p* < 0.001; SPL: F_2,33_ = 21.163, *p* < 0.001) (Figure 3A–C). These parameters gradually rose with temperature, with significant differences between groups except for SPL between the 26 °C and other groups. Q10 values of 2.12 were observed across the 26–32 °C interval but 13.82 in the 20–26 °C interval. Elevated ammonia nitrogen concentration reduced feed consumption and the number of clicks (feed consumption: F_3,44_ = 10.016, *p* < 0.001; the number of clicks: F_3,44_ = 9.813, *p* < 0.001) (Figure 3D,E). Specifically, feed consumption in the control and 4 mg/L groups was significantly higher than that in the 12 mg/L group, whereas adjacent lower concentration groups showed no significant differences. The trend in the number of clicks was consistent with that of feed consumption. SPL was less affected by ammonia nitrogen concentration (F_3,44_ = 0.187, *p* = 0.905) (Figure 3F). Elevated nitrite nitrogen concentration reduced all three parameters during feeding (feed consumption: F_3,44_ = 33.456, *p* < 0.001; the number of clicks: F_3,44_ = 10.260, *p* < 0.001; SPL: F_3,44_ = 2.873, *p* = 0.047) (Figure 3G–I). Feed consumption and the number of clicks in the control and 10 mg/L groups were significantly higher than those in the 20 mg/L and 40 mg/L groups, with the 20 mg/L group being significantly higher than the 40 mg/L group. Similarly, SPL in the control group was significantly higher than that in the 40 mg/L group.

Temporal trends in the number of clicks and SPL across all environmental factors are shown in Figure S1, with detailed inter-group and intra-group differences in acoustic characteristics presented in Tables S2 and S3.

### 3.2. The Influence of Environmental Factors on the Feeding Behavior of P. vannamei

**Figure 4:**
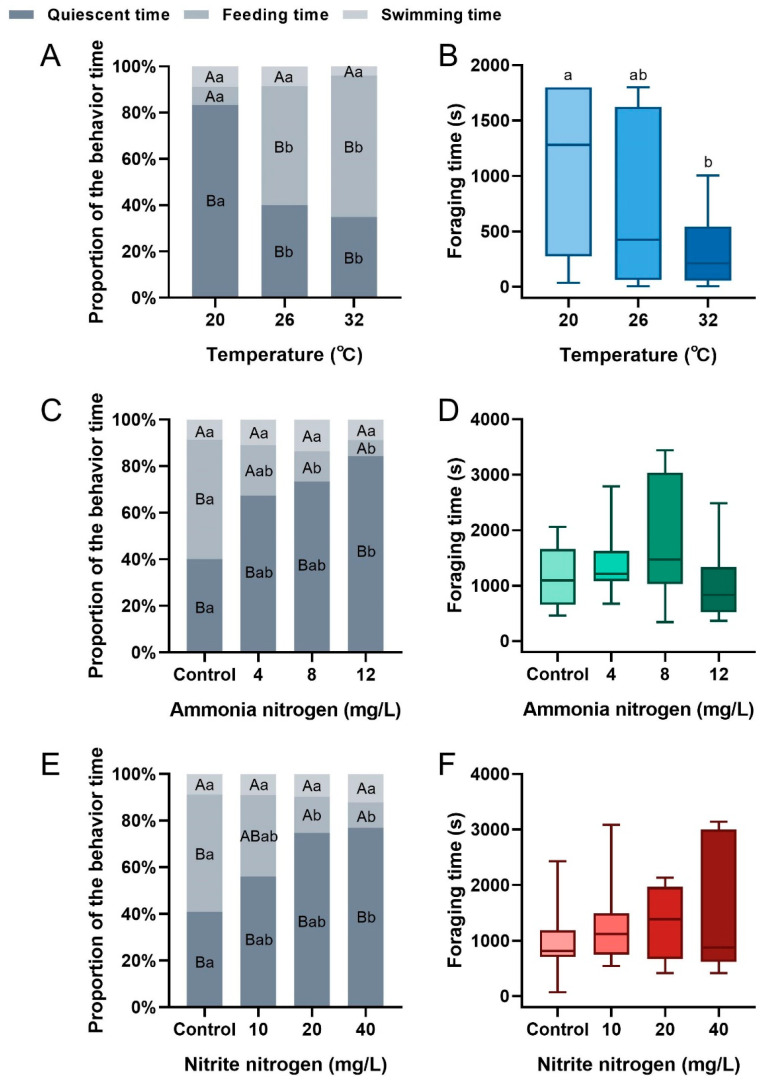
Proportion of behavior time and the foraging time of *P. vannamei* under different temperatures (**A**,**B**), ammonia nitrogen concentrations (**C**,**D**), and nitrite nitrogen concentrations (**E**,**F**). Different uppercase letters indicate significant differences between behaviors within the same treatment (*p* < 0.05), while different lowercase letters indicate significant differences in the same behavior across treatments (*p* < 0.05).

The behavioral proportions of *P. vannamei* significantly varied under different temperature conditions (20 °C treatment: F_2,33_ = 122.933, *p* < 0.001; 26 °C treatment: F_2,33_ = 6.151, *p* = 0.005; 32 °C treatment: F_2,33_ = 10.768, *p* < 0.001) (Figure 4A). As temperature was increased, the proportions of both quiescent and swimming time decreased, while feeding time increased. Significant differences in feeding and quiescent time were observed between 20 °C and higher temperatures (feeding time: F_2,33_ = 9.626, *p* = 0.001; quiescent time: F_2,33_ = 8.429, *p* = 0.001). Foraging time showed a decreasing trend with increasing temperature, with significant differences between 20 °C and 32 °C (F_2,33_ = 4.304, *p* = 0.022) (Figure 4B). Under various ammonia nitrogen concentrations, differences in feeding behavior proportions were also observed (control: F_3,44_ = 5.513, *p* = 0.009; 4 mg/L treatment: F_3,44_ = 9.209, *p* = 0.001; 8 mg/L treatment: F_3,44_ = 36.915, *p* < 0.001; 12 mg/L treatment: F_3,44_ = 34.127, *p* < 0.001) (Figure 4C). As ammonia nitrogen concentration increased, the proportion of quiescent time increased, while feeding time decreased. Compared to the control, feeding time was significantly lower in the 8 mg/L and 12 mg/L treatments (F_3,44_ = 4.190, *p* = 0.011), and the quiescent time was significantly higher in the 12 mg/L treatment (F_3,44_ = 3.807, *p* = 0.016). Foraging time was not significantly affected by ammonia nitrogen concentration but showed a trend of first increasing and then decreasing (F_3,44_ = 0.893, *p* = 0.452) (Figure 4D). Similarly, the behavioral proportions of shrimp differed under various nitrite nitrogen concentrations (control: F_3,44_ = 6.151, *p* = 0.005; 10 mg/L treatment: F_3,44_ = 23.293, *p* < 0.001; 20 mg/L treatment: F_3,44_ = 62.732, *p* < 0.001; 40 mg/L treatment: F_3,44_ = 182.078, *p* < 0.001) (Figure 4E). As nitrite nitrogen concentration increased, the proportions of both quiescent and swimming time increased, while the feeding time decreased. The feeding time proportion in the control group was significantly higher than that in the 20 mg/L and 40 mg/L treatments (F_3,44_ = 7.925, *p* < 0.001), and the quiescent time in the control group was significantly higher than that in the 40 mg/L treatment (F_3,44_ = 7.156, *p* = 0.001). Nitrite nitrogen concentration had no significant effect on the foraging time (F_3,44_ = 2.594, *p* = 0.064) (Figure 4F).

**Figure 5:**
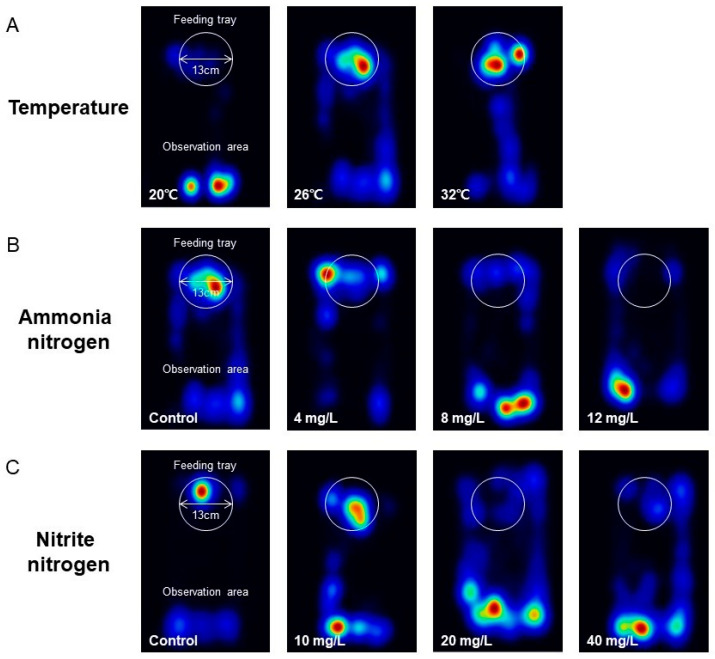
Heatmaps of feeding trajectories of *P. vannamei* under different temperature (**A**), ammonia nitrogen concentration (**B**), and nitrite nitrogen concentration (**C**) conditions, with darker shades indicating longer durations of stay.

The movement trajectories of *P. vannamei* significantly varied across different temperatures (Figure 5A). At 20 °C, shrimp tended to remain near the initial location with limited movement to the feeding tray. In contrast, at 26 °C and 32 °C, shrimp spent more time at the feeding tray, with shrimp at 32 °C showing reduced movement outside the tray. Similarly, shrimp movement trajectories differed under various ammonia nitrogen concentrations (Figure 5B). Specifically, shrimp in the 8 mg/L and 12 mg/L groups primarily stayed at the initial location with limited access to the feeding tray, whereas those in natural seawater and the 4 mg/L group stayed more at the feeding tray. Under different nitrite nitrogen concentrations, shrimp trajectories also showed distinct patterns (Figure 5C). Shrimp in the 20 mg/L and 40 mg/L groups primarily remained at the initial location, with limited movement to the feeding tray, whereas those in natural seawater and the 10 mg/L group spent more time at the feeding tray.

### 3.3. Comprehensive Analysis of Environmental Factors and Their Biological Response Variables on the Feeding Behavior of P. vannamei

**Figure 6:**
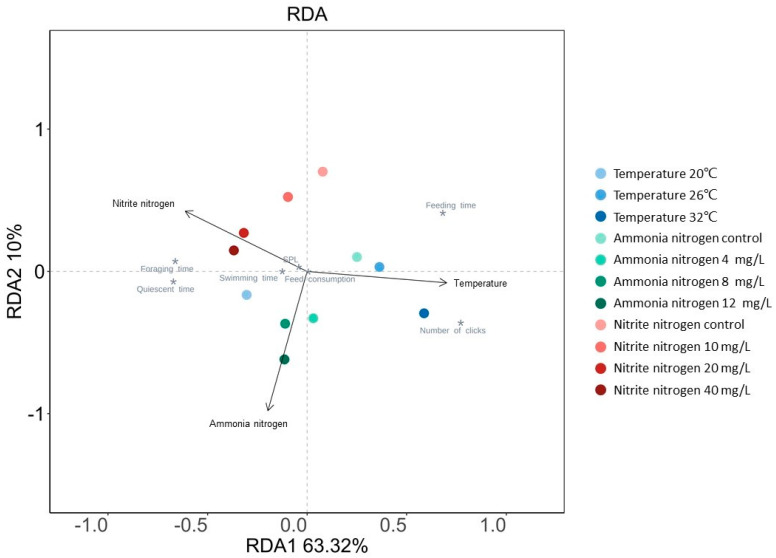
Redundancy analysis (RDA) of the effects of temperature, ammonia nitrogen concentration, and nitrite nitrogen concentration on feed consumption, acoustic feeding signals, and feeding behavior of *P. vannamei*. The arrows represent explanatory variables, and * represent biological response variables.

The RDA analysis revealed that the RDA1 axis (63.32%) and RDA2 axis (10%) together explained 73.32% of the total variance in the data (Figure 6). The arrow representing temperature exhibited the smallest angle with the RDA1 axis, and its length and projection length on the RDA1 axis were both greater than the arrows representing ammonia nitrogen and nitrite nitrogen. This indicated that temperature had the strongest comprehensive influence on the biological response variables of shrimp. Feeding time was positively correlated with temperature and negatively correlated with ammonia nitrogen and nitrite nitrogen. The number of clicks was also positively correlated with temperature and negatively correlated with nitrite nitrogen. In contrast, foraging time, quiescent time, swimming time, and SPL were primarily positively correlated with ammonia nitrogen and nitrite nitrogen and negatively correlated with temperature. The key feeding behavior parameters, such as foraging time, swimming time, quiescent time, and feeding time, had the smallest angles with the extended line of the arrow representing temperature, indicating that these behaviors were most significantly influenced by temperature. The number of clicks and SPL were most significantly affected by nitrite nitrogen. No significant difference was observed in the influence of the three environmental factors on feed consumption.

## 4. Discussion

In recent years, PAM has attracted considerable attention as an emerging monitoring technique for assessing feed intake, feed preference, and attractant performance in farmed animals, particularly in *P. vannamei* [26,27,28]. However, only a few intelligent feeding systems currently incorporate temperature-dependent feeding adjustments, with the critical influence of environmental factors on shrimp feeding behavior remaining largely unaddressed in system design. In this study, we conducted a statistical analysis of feed consumption, acoustic feeding signals, and feeding behavior in *P. vannamei* under varying conditions of temperature, ammonia nitrogen, and nitrite nitrogen. Within the range of environmental factor variations tested in this study, a rise in temperature is associated with an increase in feed consumption, the number of clicks, and SPL. Conversely, elevated concentrations of ammonia nitrogen and nitrite nitrogen led to a decrease in feed consumption and the number of clicks, while exerting minimal influence on SPL. Notably, a stable correlation was observed between feed consumption and the number of clicks across different environments. Additionally, these environmental factors significantly influenced a variety of feeding-related behaviors in *P. vannamei*.

The characteristic parameters of the “click” acoustic signals emitted by shrimp during feeding, including the timing, the number of pulses, and SPL, possess considerable significance in the monitoring of feeding behavior and aquaculture management [29]. Our study revealed a notable correlation between feed consumption and the number of clicks in *P. vannamei* across different environments (Figure 2, Table S1). Compared to SPL, the number of clicks emerged as a more reliable parameter for monitoring the feeding status of shrimp. Additionally, we found that in various environments, both the number of clicks and SPL decreased as feeding duration progressed, with the highest click count proportion and peak SPL occurring within the initial 10 min (Figure S1). This observation suggests that shrimp exhibit the highest feeding frequency and intensity during this period, aligning with the findings reported by Soares et al. and Hamilton et al. [30,31]. Based on our results, future research should prioritize the number of clicks generated during the initial feeding phase of shrimp and integrate acoustic signal characteristics under complex environmental conditions to enhance the precision of intelligent feeding systems.

In aquaculture ponds, an increase in temperature within the optimal range accelerates the metabolic rate of *P. vannamei*, enhances energy demand, and stimulates feeding activity [19,32,33]. Consistent with previous studies, our research also found that as temperature rises, the feed consumption, the number of clicks, and the SPL of shrimp all exhibit an upward trend (Figure 3A–C). Furthermore, the proportion of feeding time increased, while foraging time significantly shortened (Figure 4A,B), and the trajectories of shrimp concentrated around the feeding trays (Figure 5A). Conversely, shrimp exhibited reduced swimming and feeding behavior at 20 °C (Figure 4A), which aligns with the finding of Huang et al. that *P. vannamei* subjected to low temperatures responded more sluggishly to external stimuli [18]. The classical temperature–metabolism relationship is defined by the Q10 coefficient, typically following Q10 = 2.0 [34]. This study found Q10 values of 2.12 across the 26–32 °C interval but 13.82 in the 20–26 °C interval, representing a significant deviation from the typical value in the lower temperature. This discrepancy likely arises because 20 °C falls outside the optimal metabolic range for shrimp and suppresses feeding activity [33]. These findings align with behavioral observations showing that shrimp at 20 °C failed to reach the feed tray (Figure 5A), indicating that low temperatures impact feeding more profoundly than high temperatures within the tested gradient. Therefore, close attention should be paid to water temperature fluctuations in the pond aquaculture of *P. vannamei*. By integrating temperature and PAM information, an intelligent feeding system that synergistically monitors both temperature and PAM can be devised to dynamically adjust feeding amount. Specifically, when water temperature rises, feeding amounts should be appropriately increased, and feeding strategies optimized based on real-time changes in the number of clicks to enhance feeding efficiency and growth rate of shrimp. On the contrary, when water temperature decreases, feeding amounts should be reduced to minimize feed waste and water pollution (Figure 3A, Figure 4A, and Figure 5A). However, temperature exhibits inhibitory effects on both feeding activity and growth performance in shrimp when exceeding optimal ranges [35]. Due to experimental limitations, this study did not cover the characteristics of acoustic feeding signals in *P. vannamei* under higher water temperature conditions. In subsequent research, we will broaden the scope of investigation to accurately analyze the correlation between water temperature and acoustic feeding signals and provide more evidence for the development of precise feeding technologies.

In shrimp pond aquaculture systems, unlike dissolved oxygen and salinity, which are typically maintained within stable ranges, ammonia nitrogen and nitrite nitrogen dynamically accumulate as byproducts of feed metabolism [36]. Their concentrations are notoriously difficult to regulate with real-time precision, a challenge that inherently decreases feeding efficiency in aquaculture animals [37,38]. Our study also found that as ammonia nitrogen and nitrite nitrogen concentrations increased, the feed consumption of shrimp decreased (Figure 3D,G) This was accompanied by an increase in foraging time and quiescent time, along with a reduction in feeding time (Figure 4C–F). Moreover, the number of clicks (Figure 3E,F) and SPL (Figure 3H,I) correspondingly decreased, indicating that the two nitrogen compounds inhibited the feeding of shrimp. Behavioral trajectories showed that shrimp in water with high concentrations of these nitrogen compounds were more inclined to stay at their initial locations and less likely to reach the feeding trays (Figure 5B,C). This may be due to nitrogen compounds damaging the gill tissue structure of shrimp [39], diminishing the oxygen-carrying capacity of hemocyanin [40,41]. As a result, shrimp are compelled to adjust their behavioral adaptation strategies by reducing feed intake to conserve energy and enhance survival probability [42], which is consistent with the adaptation mechanisms of fish such as common carp (*Cyprinus carpio*) [43,44]. Based on our results, neglecting the impact of inorganic nitrogen on the feeding of shrimp may lead to delayed adjustment in feeding amounts, causing the accumulation of uneaten feed, elevated ammonia nitrogen and nitrite nitrogen concentrations, and ultimately resulting in a vicious cycle of water pollution and inhibited shrimp feeding. Therefore, in future assessments of feeding strategies, it is necessary to include ammonia nitrogen and nitrite nitrogen concentrations as key information in the decision-making process of PAM feeding systems. Specifically, the system should issue warnings when inorganic nitrogen concentrations rise and correspondingly reduce feed amounts to maintain the stability of the aquaculture environment and ensure the good growth of shrimp.

Various environmental factors exert differential impacts on the feeding behavior of *P. vannamei*. Among the three environmental factors investigated in this study, temperature had the most pronounced effect on the overall biological response variables of shrimp (Figure 6). This is likely attributed to the fact that shrimp are poikilothermic and their digestive enzyme activity is modulated by temperature, thus enabling temperature to exert a direct impact on their feeding behavior [45,46]. However, ammonia nitrogen and nitrite nitrogen primarily exert their effects by impacting the respiratory, immune, and metabolic systems of aquatic organisms, indirectly reducing shrimp feeding motivation through the imposition of stress [47]. Furthermore, *P. vannamei* possesses physiological mechanisms to adapt to inorganic nitrogen stress, which to some extent attenuates the impact of nitrogen compounds on feeding behavior [48]. Consequently, in aquaculture management, it is crucial to prioritize maintaining water temperature within an appropriate physiological range to ensure the feeding behavior and metabolic efficiency of shrimp. It is important to recognize that in actual aquaculture ponds, environmental factors such as temperature, ammonia nitrogen, and nitrite nitrogen do not exist in isolation. Instead, they interact in complex ways to produce combined effects on shrimp, amplifying stress effects and increasing the complexity and challenges of feeding management [49].

Synthesizing the results of this study, the number of clicks across various aquaculture environments consistently correlates with feed consumption, accurately reflecting the feeding status of shrimp. Compared to ammonia nitrogen and nitrite nitrogen, temperature variations exert a more pronounced impact on the feed consumption, acoustic characteristics, and behavior of *P. vannamei*, emerging as the most significant environmental factor influencing its feeding. Shrimp exhibit gregarious behavior, with significant differences observed between individual and group feeding dynamics in aquaculture environments [31]. This study investigates the effects of environmental factors on the feeding behavior of *P. vannamei* at the individual level. Furthermore, we will refine analyses of group feeding behavior under environmental variations and explore the synergistic effects of multiple environmental factors on *P. vannamei* feeding performance, thereby providing a scientific basis for optimizing intelligent feeding systems.

## 5. Conclusions

This study utilizes PAM to investigate the effects of temperature, ammonia nitrogen, and nitrite nitrogen on the feeding behavior and acoustic signals of *P. vannamei*. Results reveal a significant positive correlation between the number of clicks and feed consumption, with temperature identified as the most influential environmental factor. Elevated temperatures stimulate feeding activity and acoustic signal intensity, whereas high concentrations of ammonia nitrogen and nitrite nitrogen suppress both parameters. This research is the first to quantify environmental impacts on *P. vannamei* feeding acoustics, validating the feasibility of PAM for assessing feeding status across diverse conditions. The findings underscore the necessity of integrating environmental monitoring modules into intelligent feeding systems, providing a scientific foundation for the development of precision feeding technologies and the enhancement of aquaculture efficiency.

## Figures and Tables

**Table 1:** The description of behavioral component classification of *P. vannamei*.

Behavioral Component	Description
Feeding time	The duration (in seconds) that *P. vannamei* spends feeding within the feeding tray. In EthoVision, this is defined as “the time spent within the observation area (i.e., the feeding tray).”
Quiescent time	The duration (in seconds) during which *P. vannamei* remains stationary during the experiment.
Swimming time	The duration (in seconds) that *P. vannamei* continuously swims during the experiment.
Foraging time	The duration (in seconds) taken by *P. vannamei* to first enter the feeding tray. In EthoVision, this is defined as “the time of first entry into the observation area.”

## Data Availability

The data presented in this study are available in this article. Further information is available upon request from the corresponding author.

## Supplementary Materials

The following supporting information can be downloaded at: https://www.mdpi.com/article/10.3390/ani15142113/s1, Figure S1: Trends in the number of clicks and SPL over time at different temperatures (A,B), ammonia nitrogen concentrations (C,D), and nitrite nitrogen concentrations (E,F); Table S1: Multivariate linear regression analysis of feed consumption and both the number of clicks and SPL in *Penaeus vannamei*; Table S2: Analysis of differences in acoustic signal characteristics between different treatment groups within the same time period; Table S3: Analysis of differences in acoustic signal characteristics across different time periods under the same treatment conditions.
