# Supplementary material for: Environmental Factors Modulate Feeding Behavior of Penaeus vannamei: Insights from Passive Acoustic Monitoring
Source: Animals (Basel). 2025 Jul 17;15(14):2113. doi: 10.3390/ani15142113 (PMC12291832; doi:10.3390/ani15142113)
Supplement: Supplementary file 1 [file animals-15-02113-s001.zip › animals-3655015-supplementary.pdf]

Table S1. Multivariate linear regression analysis of feed consumption and both the number of clicks and SPL in *Penaeus vannamei.*

Environmental factor	Variable	β	*t*	*P*	95.0% CI	
					Lower limit	Upper limit
Temperature	Intercept		-3.724	0.001	-7.421	-2.178
	The number of clicks	0.601	6.248	**0.000**	0.000	0.000
	SPL	0.393	4.089	**0.000**	0.030	0.089
Ammonia nitrogen	Intercept		0.415	0.680	-0.787	1.195
	The number of clicks	0.797	9.019	**0.000**	0.000	0.000
	SPL	0.068	0.766	0.448	-0.007	0.015
Nitrite nitrogen	Intercept		1.399	0.169	-0.436	2.418
	The number of clicks	0.801	6.224	**0.000**	0.000	0.000
	SPL	-0.106	-0.825	0.414	-0.023	0.010

Note: The bolded factors have a significant impact on feed consumption ($P < 0.05$).

Under varying conditions of temperature, ammonia nitrogen concentration, and nitrite nitrogen concentration, a significant positive correlation was observed between the number of clicks and feed consumption in *P. vannamei* (Temperature: β=0.601, $P < 0.001$; ammonia nitrogen: β=0.797, $P < 0.001$; nitrite nitrogen: β=0.801, $P < 0.001$). In contrast, SPL demonstrated a significant positive correlation with feed consumption only under different temperature (β=0.393, $P < 0.001$), while no significant relationship was found between SPL and feed consumption under varying ammonia nitrogen (β=0.068, $P = 0.448$) or nitrite nitrogen conditions (β=–0.106, $P = 0.414$).

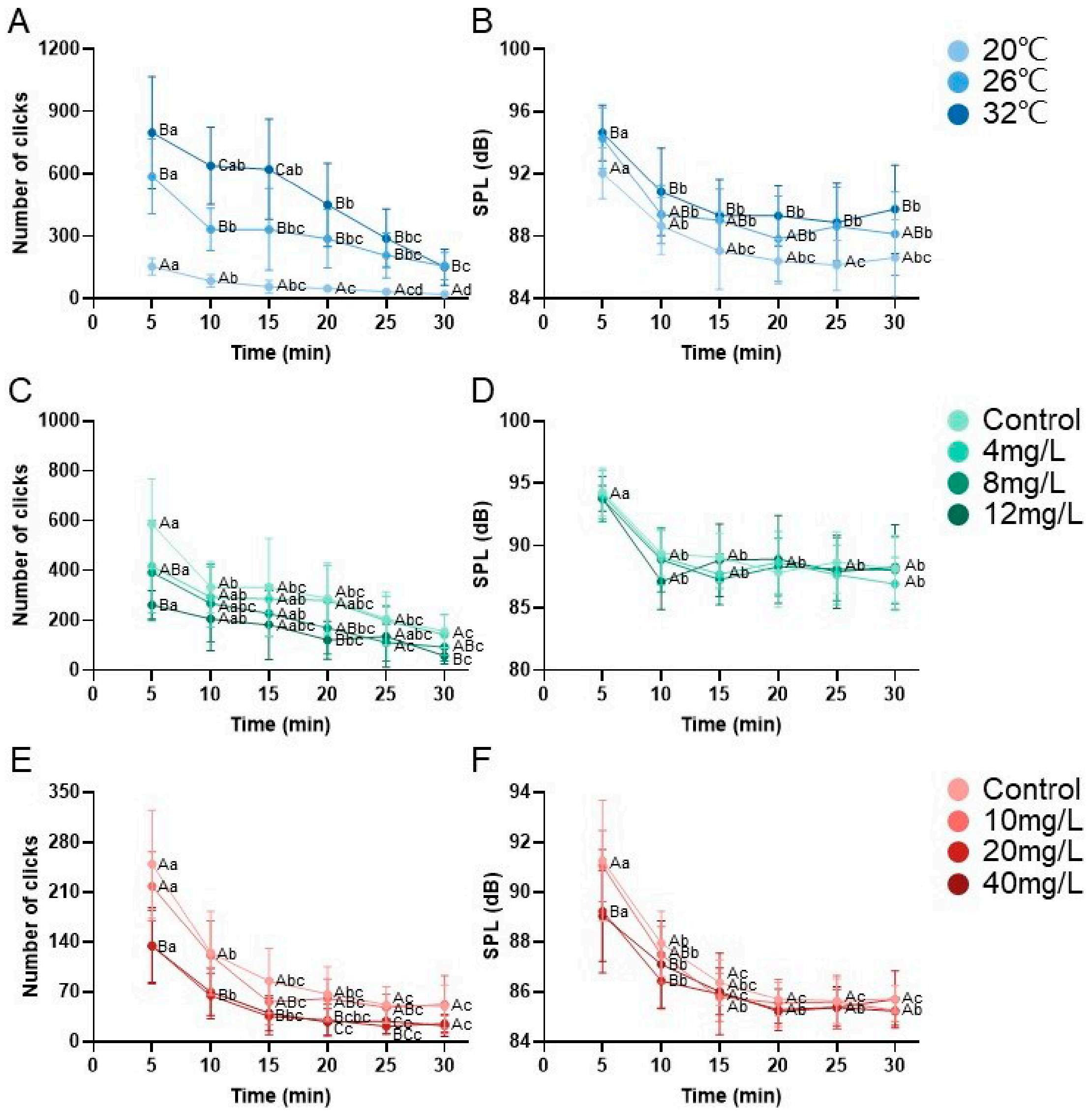

Figure S1. Trends in the number of clicks and SPL over time at different temperatures (A-B), ammonia nitrogen concentrations (C-D), and nitrite nitrogen concentrations (E-F). Different uppercase letters indicate significant differences in acoustic signal characteristics between different treatment groups within the same time period ($P < 0.05$); different lowercase letters indicate significant differences in acoustic signal characteristics between different time periods under the same treatment conditions ($P < 0.05$).

Under different temperatures, both the number of clicks (Fig. S1A) and SPL (Fig. S1B) in *P. vannamei* showed a decreasing trend as feeding time increased, with significant differences observed across time periods. In the 20°C, 26°C, and 32°C treatment groups, the number of clicks in the first 10 minutes accounted for 60.96 ± 6.78%, 49.68 ± 8.89%, and 48.82 ± 6.73% of the total, respectively. In varying ammonia nitrogen concentrations, both the number of clicks (Fig. S1C) and SPL (Fig. S1D) decreased as feeding time increased. The number of clicks showed significant differences across time periods, while the SPL did not. In the control group and the 4 mg/L, 8 mg/L, and 12 mg/L treatment groups, the number of clicks in the first 10 minutes accounted for 49.68 ± 8.89%, 44.13 ± 9.38%, 51.05 ± 11.38%, and 50.45 ± 14.78% of the total, respectively. In different nitrite nitrogen concentrations, both the number of clicks (Fig. S1E) and SPL (Fig. S1F) exhibited a downward trend over time. The number of clicks did not show significant differences only between the 25-30 minute period, while the SPL showed significant differences between the first 5

minutes and the 5-10 minute period. In the control group and the 10 mg/L, 20 mg/L, and 40 mg/L treatment groups, the number of clicks in the first 10 minutes accounted for 60.43 ± 6.27%, 63.54 ± 3.94%, 65.98 ± 8.41%, and 64.59 ± 8.12% of the total, respectively. The detailed results of the significance analysis of differences for this figure are presented in Tables S2 and S3.

Table S2. Analysis of differences in acoustic signal characteristics between different treatment groups within the same time period.

Treatments	Response variable	F-statistic	*P*-value
	**Temperature**		
20°C	Number of clicks	$F_{5,66} = 42.069$	$P < 0.001$
	SPL	$F_{5,66} = 15.911$	$P < 0.001$
26°C	Number of clicks	$F_{5,66} = 13.758$	$P < 0.001$
	SPL	$F_{5,66} = 12.751$	$P < 0.001$
32°C	Number of clicks	$F_{5,66} = 17.936$	$P < 0.001$
	SPL	$F_{5,66} = 9.706$	$P < 0.001$
	**Ammonia nitrogen**		
Control	Number of clicks	$F_{5,66} = 13.758$	$P < 0.001$
	SPL	$F_{5,66} = 12.751$	$P < 0.001$
4 mg/L	Number of clicks	$F_{5,66} = 7.132$	$P < 0.001$
	SPL	$F_{5,66} = 18.332$	$P < 0.001$
8 mg/L	Number of clicks	$F_{5,66} = 10.045$	$P < 0.001$
	SPL	$F_{5,66} = 12.101$	$P < 0.001$
12 mg/L	Number of clicks	$F_{5,66} = 6.062$	$P < 0.001$
	SPL	$F_{5,66} = 8.572$	$P < 0.001$
	**Nitrite nitrogen**		
Control	Number of clicks	$F_{5,66} = 28.974$	$P < 0.001$
	SPL	$F_{5,66} = 35.414$	$P < 0.001$
10 mg/L	Number of clicks	$F_{5,66} = 37.964$	$P < 0.001$
	SPL	$F_{5,66} = 55.238$	$P < 0.001$
20 mg/L	Number of clicks	$F_{5,66} = 25.554$	$P < 0.001$
	SPL	$F_{5,66} = 12.553$	$P < 0.001$
40 mg/L	Number of clicks	$F_{5,66} = 25.564$	$P < 0.001$
	SPL	$F_{5,66} = 19.212$	$P < 0.001$

Table S3. Analysis of differences in acoustic signal characteristics across different time periods under the same treatment conditions.

Response variable	Time	F-statistic	*P*-value
	**Temperature**		
Number of clicks	5 min	$F_{2,33} = 36.388$	$P < 0.001$
	10 min	$F_{2,33} = 60.590$	$P < 0.001$
	15 min	$F_{2,33} = 29.481$	$P < 0.001$
	20 min	$F_{2,33} = 24.528$	$P < 0.001$
	25 min	$F_{2,33} = 19.653$	$P < 0.001$
	30 min	$F_{2,33} = 17.366$	$P < 0.001$
SPL	5 min	$F_{2,33} = 7.388$	$P = 0.002$
	10 min	$F_{2,33} = 3.041$	$P = 0.061$
	15 min	$F_{2,33} = 3.494$	$P = 0.042$
	20 min	$F_{2,33} = 5.738$	$P = 0.007$
	25 min	$F_{2,33} = 5.546$	$P = 0.008$
	30 min	$F_{2,33} = 4.140$	$P = 0.025$

	**Ammonia nitrogen**		
Number of clicks	5 min	$F_{3,44} = 7.997$	$P < 0.001$
	10 min	$F_{3,44} = 2.026$	$P = 0.124$
	15 min	$F_{3,44} = 2.957$	$P = 0.043$
	20 min	$F_{3,44} = 5.850$	$P = 0.002$
	25 min	$F_{3,44} = 2.559$	$P = 0.067$
	30 min	$F_{3,44} = 6.306$	$P = 0.001$
SPL	5 min	$F_{3,44} = 0.266$	$P = 0.849$
	10 min	$F_{3,44} = 2.466$	$P = 0.075$
	15 min	$F_{3,44} = 1.952$	$P = 0.135$
	20 min	$F_{3,44} = 0.330$	$P = 0.804$
	25 min	$F_{3,44} = 0.315$	$P = 0.815$
	30 min	$F_{3,44} = 0.609$	$P = 0.613$
	**Nitrite nitrogen**		
Number of clicks	5 min	$F_{3,44} = 12.138$	$P < 0.001$
	10 min	$F_{3,44} = 6.268$	$P = 0.001$
	15 min	$F_{3,44} = 5.502$	$P = 0.003$
	20 min	$F_{3,44} = 6.457$	$P = 0.001$
	25 min	$F_{3,44} = 6.816$	$P = 0.001$
	30 min	$F_{3,44} = 3.978$	$P = 0.014$
SPL	5 min	$F_{3,44} = 3.787$	$P = 0.017$
	10 min	$F_{3,44} = 2.636$	$P = 0.061$
	15 min	$F_{3,44} = 0.573$	$P = 0.636$
	20 min	$F_{3,44} = 1.098$	$P = 0.360$
	25 min	$F_{3,44} = 0.275$	$P = 0.843$
	30 min	$F_{3,44} = 1.651$	$P = 0.191$
